# Family Nutrition and Physical Activity Practices Associated with Overweight and Obesity in Children: A Cross-Sectional Study

**DOI:** 10.3390/children13010123

**Published:** 2026-01-14

**Authors:** Emine Zahide Özdemir, Murat Bektaş

**Affiliations:** Pediatric Nursing, Faculty of Nursing, Dokuz Eylul University, Izmir 35390, Türkiye

**Keywords:** childhood obesity, overweight, family nutrition and physical activity (FNPA), feeding behavior, physical activity, screen time, family environment

## Abstract

**Highlights:**

**What are the main findings?**

• Healthier beverage choices, family meals, and eating habits predict lower obesity risk in children.
• Screen exposure significantly increases obesity odds.

**What are the implications of the main findings?**

• Pediatric professionals should promote structured family meals and limit screen time.
• Family-centered interventions may effectively prevent childhood obesity.

**Abstract:**

**Background/Objectives**: Childhood overweight and obesity are influenced by family-level behaviors related to nutrition, physical activity, and daily routines. This study aimed to In contrast to screen time family nutrition and physical activity practices for overweight and obesity among children aged 6–17 years in Türkiye. **Methods**: A cross-sectional study was conducted with 214 children recruited from a community setting. Sociodemographic data and anthropometric measurements were collected, and family practices were assessed using the Family Nutrition and Physical Activity Scale–Turkish version (FNPA-TR). Binary logistic regression analyses were performed separately for overweight and obesity outcomes. **Results**: Healthier beverage choices were the only significant predictor of overweight, reducing the odds by 62%. Obesity was predicted by three FNPA domains: family meal frequency, family eating habits, and screen time. Frequent family meals and healthier eating habits were associated with lower obesity risk, whereas higher screen exposure increased the likelihood of obesity. **Conclusions**: Beverage choices, family meal patterns, eating habits, and screen exposure emerged as key behavioral predictors of unhealthy weight status in children. These findings highlight key family-centered prevention targets for pediatric nursing and public health, including promoting healthy beverage consumption, strengthening structured family eating routines, and reducing screen exposure in children.

## 1. Introduction

Childhood overweight and obesity have become major public health concerns worldwide, with rising prevalence rates observed in many different socioeconomic and geographic settings [1,2,3]. Excess body weight during childhood is associated with a wide range of adverse health outcomes, including metabolic disorders, cardiovascular complications, impaired psychosocial functioning, and reduced quality of life. Moreover, obesity that begins in childhood often persists into adulthood, creating a long-term disease burden for individuals, families, and health systems [4,5]. National and hospital-based studies from Türkiye indicate that obesity affects approximately 21–32% of children and adolescents aged 6–17 years, underscoring the magnitude of the problem and the urgent need to identify modifiable family- and environment-related risk factors [6,7]. Although the core determinants of childhood obesity, such as unhealthy dietary patterns, physical inactivity, and excessive screen exposure, are globally recognized, their relative contribution and expression may vary across countries. In Türkiye, rapid urbanization, the shift away from traditional family meal patterns, increased consumption of convenience foods, and prolonged screen time have emerged alongside a strong family-centered caregiving structure, potentially shaping obesity risk in distinct ways.

A growing body of evidence suggests that childhood obesity is shaped by multiple interacting determinants, many of which originate within the family context. Parents influence children’s eating and physical activity patterns through food availability, meal structure, beverage options, household routines, and modeling of healthy or unhealthy behaviors [8,9,10,11]. Family meals, nutrition habits, beverage choices, sedentary screen behaviors, and opportunities for physical activity have all been independently associated with children’s weight status [12,13]. Because these behaviors occur within the home, they represent modifiable and culturally sensitive targets for early prevention.

Family-related behaviors such as shared meals, overall eating patterns, beverage consumption, screen exposure, physical activity routines, and sleep schedules are increasingly recognized as central determinants of childhood overweight and obesity [14,15]. Although numerous studies have shown that these practices cluster within families and shape children’s weight trajectories, the evidence is often fragmented, with individual behaviors examined in isolation and without a comprehensive framework [16,17,18]. In Türkiye, empirical work on how constellations of family nutrition and physical activity practices relate to children’s weight status is still limited, and few studies have evaluated which specific practices (for example, sugary beverage consumption or screen time) are more strongly associated with overweight versus obesity. Moreover, most existing research is based on school samples, which may not fully capture the behaviors of families in everyday community settings [19,20]. Community-based investigations are needed to provide a more realistic picture of children’s daily exposure to unhealthy lifestyle conditions and to identify the most salient family-level targets for intervention. From an international perspective, examining family nutrition and physical activity patterns in a middle-income country undergoing rapid lifestyle transition contributes to a more nuanced understanding of how sociocultural context influences obesity-related behaviors. Evidence from non-Western settings such as Türkiye remains limited, and studies addressing this gap may enhance the cross-cultural applicability of family-based obesity prevention frameworks.

Given these gaps, there is a clear need for research exploring which specific family practices are most strongly associated with unhealthy weight status among Turkish children. Understanding these relationships can help pediatric nurses and other healthcare professionals design targeted, family-centered interventions that address the most influential behavioral patterns. At the national level, identifying modifiable family-based behaviors related to nutrition, physical activity, and screen use is particularly relevant for informing preventive strategies in primary care and community settings in Türkiye. Such evidence may support pediatric nurses, family health professionals, and policymakers in prioritizing family-centered approaches to childhood obesity prevention.

Therefore, the present study aimed to examine the predictive value of family nutrition and physical activity practices—measured using the FNPA-TR—for overweight and obesity among children 6–17 years in Türkiye. Logistic regression analyses were conducted to determine which family-level behaviors best predicted (1) overweight and (2) obesity status based on BMI percentiles specific to Turkish children. The findings are expected to contribute to the pediatric nursing literature by identifying concrete, modifiable behaviors that can inform culturally appropriate prevention strategies.

## 2. Materials and Methods

This study employed a descriptive, correlational and cross-sectional design to examine the predictive value of family nutrition and physical activity practices for children’s overweight and obesity status.

### 2.1. Participants

The study was conducted in Balçova, a predominantly urban district of İzmir, the third-largest metropolitan city in Türkiye. Participants were recruited from a shopping center, which serves families from diverse socioeconomic backgrounds and functions as a common public space for routine family activities. The study group consisted of 214 children aged 6–17 years (M = 11.08, SD = 3.60) who visited the shopping center during lunchtime. Participants were recruited through voluntary participation.

The required sample size was calculated using the G*Power 3.1 program (Heinrich Heine University Düsseldorf, Düsseldorf, Germany), in binary logistic regression analysis, assuming a medium effect, 80% power, and a significance level of 0.05, which indicated a minimum sample size of 210 participants. Although the minimum required number was 210, data collection continued until the end of the planned study period, resulting in a sample size (N = 214). This increased sample size further enhanced the statistical power and robustness of the findings.

### 2.2. Inclusion and Exclusion Criteria

Children were eligible to participate if they were between 6 and 17 years of age, able to understand Turkish, and agreed to participate with parental consent. Children and parents who did not sign the informed consent form or withdrew during data collection were excluded from the study.

### 2.3. Ethical Considerations

The study was conducted in accordance with the Declaration of Helsinki and approved by the Dokuz Eylul University Non-Interventional Research Ethics Committee (Approval No: 2024/29-12). Written informed consent was obtained from all parents, and verbal assent was obtained from the children.

### 2.4. Data Collection Tools

#### 2.4.1. Sociodemographic Information Form

This form included questions about age, gender, height, and weight. Height and weight were measured using standardized equipment, and Body Mass Index (BMI) was calculated as weight (kg) divided by height squared (m^2^).

#### 2.4.2. Family Nutrition and Physical Activity Scale (FNPA-TR)

It was used to assess family-based behavioral and environmental factors associated with children’s risk for overweight and obesity. The original FNPA tool was developed by Ihmels et al. to screen family practices contributing to children’s weight gain, and its predictive validity for childhood overweight and obesity has been well documented [10]. The Turkish adaptation and validation of the scale was conducted by Ekici et al., demonstrating acceptable psychometric properties for use among Turkish families [19].

The FNPA-TR consists of 20 items across 10 subscales, each representing modifiable family practices: (1) family meals, (2) family eating habits, (3) food choices, (4) beverage choices, (5) restriction/reward, (6) screen time, (7) healthy environment, (8) family physical activity, (9) child physical activity, (10) sleep routines. Each item is rated on a 4-point Likert scale (1 = never/almost never, 4 = very often/always). Six items are reverse-scored to ensure that higher scores consistently reflect healthier family practices. Total scale scores range from 20 to 80, with higher scores indicating a family environment that supports healthy nutrition, adequate physical activity, and lower risk for unhealthy weight gain. The scale has no clinical cut-off, and total scores are treated as continuous predictors. In the Turkish adaptation study, FNPA-TR demonstrated good internal consistency (Cronbach’s α = 0.72) and strong test–retest reliability (ICC range: 0.42–0.93), confirming its suitability for assessing obesogenic family environments. In the current study, FNPA-TR subscale scores and the total score were used as independent variables in logistic regression models predicting overweight and obesity status in children 6–17 years. In the current study, the FNPA-TR demonstrated excellent internal consistency (Cronbach’s alpha = 0.95).

### 2.5. Data Collection Procedure

Data collection was conducted by trained pediatric nurses with experience in pediatric health. The study was conducted in İzmir, Türkiye, and participants were recruited from a shopping mall located in the Balçova district, which predominantly serves an urban population. After obtaining ethical and institutional permissions, data collection was conducted between September and October 2024. Children and parents were approached in the shopping center’s food court area, informed about the study purpose, and invited to participate. The FNPA questionnaire was completed by parents on behalf of their children, including both children and adolescents. Anthropometric measurements were performed on-site, and questionnaires were completed in approximately 15 min. Anthropometric measurements were obtained during data collection sessions at the recruitment site. The exact time of day was not systematically recorded. Height was measured using a calibrated portable stadiometer with children standing upright, barefoot, and with the head positioned in the Frankfurt plane (eyes looking straight ahead). Weight was measured using a digital scale with children wearing light clothing and no shoes, and values were recorded to the nearest 0.1 kg. BMI was calculated as weight (kg) divided by height squared (m^2^).

### 2.6. Data Analysis

Data were analyzed using IBM SPSS Statistics 26.0 (IBM Corp., Armonk, NY, USA). Descriptive statistics (frequency, percentage, mean, standard deviation) were used to summarize sociodemographic characteristics, anthropometric measurements, BMI categories, and FNPA-TR scores. The internal consistency of the FNPA-TR was examined using Cronbach’s alpha coefficient. Overweight and obesity were defined using standard BMI-for-age percentile cut-offs, in which values ≥85th percentile indicated overweight and ≥95th percentile indicated obesity, consistent with international CDC criteria [21]. Age- and sex-specific BMI percentiles based on the reference values established by Neyzi et al. were used to classify children as underweight, normal weight, overweight, or obese [22]. To examine the predictive value of FNPA-TR subscale scores for weight status, binary logistic regression analyses were conducted separately for overweight (0 = not overweight, 1 = overweight) and obesity (0 = not obese, 1 = obese). Given the wide age range and developmental differences in eating autonomy, all analyses were stratified by age group (children < 12 years; adolescents ≥ 12 years). Age-stratified multivariable logistic regression models were estimated, and subgroup results are presented in Tables 2 and 3. Before the regression analysis, multicollinearity was assessed using the Variance Inflation Factor (VIF) and tolerance. For all predictors, the VIF was found to be less than 10, and tolerance values were greater than 0.1. No multicollinearity was detected. A significance level of *p* < 0.05 was adopted for all statistical tests.

## 3. Results

### 3.1. Participant Characteristics

Participant characteristics are presented in Table 1. A total of 214 children aged 6–17 years participated in the study; 123 participants were children (<12 years), and 91 were adolescents (≥12 years). The mean age of the sample was 11.08 ± 3.6 years. The average height was 145.8 ± 20.9 cm, and the average weight was 43.1 ± 18.1 kg. More than half of the children were female (57.5%), and 11.7% had a chronic illness. BMI categories were derived from national reference growth charts for Turkish children and adolescents [22]. Accordingly, 66.8% of the children were classified as having normal weight, 14% as overweight, 12.1% as obese, and 7% as underweight (Table 1).

**Table 1:** Sociodemographic and clinical characteristics of the participants (*n* = 214).

Variable	n (%)	M ± SD
Child characteristics		
Age (years)	214	11.08 ± 3.6
Children (<12 years)	123	8.4 ± 1.8
Adolescents (≥12 years)	91	14.7 ± 1.8
Sex		
Female	123 (57.5)	
Male	91 (42.5)	
Height (cm)	214	145.8 ± 20.9
Weight (kg)	214	43.1 ± 18.1
BMI	214	19.38 ± 4.48
BMI category ^a^		
Underweight	15 (7)	
Normal weight	143 (66.8)	
Overweight	30 (14)	
Obese	26 (12.1)	
Chronic illness		
Yes	25 (11.7)	
No	189 (88.3)	
Family and socioeconomic characteristics		
Maternal education level		
Primary school or less	42 (19.6)	
Secondary school	58 (27.1)	
High school	61 (28.5)	
University or higher	53 (24.8)	
Paternal education level		
Primary school or less	49 (22.9)	
Secondary school	63 (29.4)	
High school	55 (25.7)	
University or higher	47 (22.0)	
Maternal employment status		
Employed	98 (45.8)	
Not employed	116 (54.2)	
Household monthly income *		
Low	64 (29.9)	
Middle	102 (47.7)	
High	48 (22.4)	
Number of children in household		1.9 ± 0.8
Lifestyle and family environment		
FNPA total score	214	55.5 ± 14.1

M = Mean; SD = Standard Deviation. ^a^ BMI percentiles were calculated according to national reference growth charts for Turkish children and adolescents. FNPA: Family Nutrition and Physical Activity; higher scores indicate healthier family environments. * Household income categories were defined according to self-reported parental information.

### 3.2. Predictors of Overweight Status

Results of the logistic regression analysis predicting overweight status are shown in Table 2. Binary logistic regression was conducted to identify predictors of being overweight. As shown in Table 2, beverage choices emerged as the only significant predictor of overweight status. Specifically, healthier beverage choices were associated with a 62% reduction in the odds of being overweight (OR = 0.378, 95% CI = 0.204–0.700, *p* = 0.002). Age-stratified analyses revealed limited variability and reduced statistical power for several FNPA subscales in the adolescent group; therefore, regression estimates could not be reliably calculated for these predictors and are indicated as not estimated in Table 2.

**Table 2:** Binary logistic regression analyses predicting overweight status in the total sample and stratified by age group.

Predictor	Total Sample (*n* = 214) OR (95% CI)	*p*-Value	Children (<12 years) (*n* = 197) OR (95% CI)	*p*-Value	Adolescents (≥12 years) (*n* = 88) OR (95% CI)	*p*-Value
Family Meal Frequency	1.62 (0.88–2.97)	0.121	1.32 (0.75–2.31)	0.341	-	-
Family Eating Habits	1.40 (0.82–2.38)	0.214	1.02 (0.62–1.69)	0.938	-	-
Food Choices	0.69 (0.40–1.21)	0.197	0.78 (0.45–1.34)	0.365	-	-
Beverage Choices	**0.38 (0.20–0.70)**	**0.002**	**0.55 (0.32–0.95)**	**0.032**	**0.19 (0.05–0.70)**	**0.012**
Restriction/Reward	0.90 (0.56–1.45)	0.672	0.79 (0.51–1.23)	0.296	-	-
Screen Time	0.69 (0.40–1.21)	0.196	0.81 (0.48–1.35)	0.409	0.65 (0.36–1.21)	0.174
Healthy Environment	0.78 (0.45–1.36)	0.381	1.00 (0.53–1.88)	0.997	-	-
Family Physical Activity	0.67 (0.40–1.13)	0.135	0.88 (0.50–1.56)	0.663	-	-
Child Physical Activity	0.90 (0.51–1.57)	0.701	0.80 (0.44–1.46)	0.473	-	-
Sleep Routines	0.69 (0.41–1.16)	0.159	0.71 (0.40–1.24)	0.229	-	-

OR: Odds Ratio; CI: Confidence Interval. Binary logistic regression models were adjusted for all listed family nutrition and physical activity variables. Overweight was defined as BMI-for-age between the 85th and <95th percentiles; reference group: normal weight (<85th percentile). Children: <12 years; Adolescents: ≥12 years. In adolescents, multivariable models were estimated using a reduced set of predictors to ensure model stability. Bold values indicate statistical significance (*p* < 0.05).

### 3.3. Predictors of Obesity Status

Table 3 presents the logistic regression analysis for obesity status. A second logistic regression analysis examined predictors of obesity (Table 3). Three FNPA domains were significantly associated with obesity status: Family meal frequency was inversely associated with obesity (OR = 0.477, 95% CI = 0.235–0.965, *p* = 0.040). Family eating habits also significantly predicted lower obesity risk (OR = 0.486, 95% CI = 0.245–0.964, *p* = 0.039). Screen time was a strong predictor, with longer screen exposure increasing the likelihood of obesity; healthier (lower) screen time scores reduced the odds by 56% (OR = 0.441, 95% CI = 0.222–0.875, *p* = 0.019). The remaining FNPA subscales—food choices, beverage choices, restriction/reward, healthy environment, family physical activity, child physical activity, and sleep routines—did not significantly predict obesity (*p* > 0.05). Age-stratified analyses showed that associations were generally stronger and more consistent among children (<12 years), whereas estimates in adolescents were less stable and did not reach statistical significance for most predictors, likely due to smaller subgroup size and increased behavioral autonomy. Table 2 and Table 3 present the regression results for the total sample and stratified by age group. Associations showed partially different patterns between children and adolescents.

**Table 3:** Binary logistic regression analyses predicting obesity status in the total sample and stratified by age group.

Predictor	Total Sample (*n* = 214) OR (95% CI)	*p*-Value	Children (<12 year) (*n* = 197) OR (95% CI)	*p*-Value	Adolescents (≥12 year) (*n* = 88) OR (95% CI)	*p*-Value
Family Meal Frequency	**0.48 (0.24–0.97)**	**0.040**	0.49 (0.21–1.14)	0.096	0.36 (0.06–2.29)	0.279
Family Eating Habits	**0.49 (0.25–0.96)**	**0.039**	0.48 (0.23–1.01)	0.053	0.62 (0.18–2.16)	0.456
Food Choices	0.94 (0.50–1.77)	0.850	0.88 (0.32–2.42)	0.799	1.40 (0.25–7.92)	0.706
Beverage Choices	1.51 (0.80–2.86)	0.203	1.32 (0.60–2.91)	0.498	3.12 (0.33–29.84)	0.323
Restriction/Reward	0.83 (0.47–1.45)	0.505	0.88 (0.49–1.59)	0.669	2.63 (0.43–16.18)	0.298
Screen Time	**0.44 (0.22–0.88)**	**0.019**	**0.26 (0.09–0.76)**	**0.013**	0.20 (0.03–1.41)	0.106
Healthy Environment	0.97 (0.50–1.85)	0.917	0.80 (0.35–1.83)	0.597	0.61 (0.08–4.56)	0.628
Family Physical Activity	0.85 (0.46–1.57)	0.598	**0.42 (0.19–0.94)**	**0.034**	5.58 (0.52–60.09)	0.156
Child Physical Activity	0.99 (0.52–1.89)	0.971	0.92 (0.35–2.43)	0.861	0.17 (0.01–2.94)	0.222
Sleep Routines	0.81 (0.46–1.42)	0.453	0.85 (0.35–2.10)	0.728	0.41 (0.12–1.39)	0.152

OR: Odds Ratio; CI: Confidence Interval. Binary logistic regression models were adjusted for all listed family nutrition and physical activity variables. Overweight was defined as BMI-for-age between the 85th and <95th percentiles; reference group: normal weight (<85th percentile). Children: <12 years; Adolescents: ≥12 years. Bold values indicate statistical significance (*p* < 0.05).

## 4. Discussion

This study investigated the predictive value of family nutrition and physical activity practices for overweight and obesity among children 6–17 years. Overall, the findings highlight the prominent role of family-level behavioral patterns—particularly beverage consumption, family mealtime practices, eating habits, and screen use—in shaping children’s weight status. These results align with the ecological perspective suggesting that child health behaviors are strongly embedded within family routines and home environments [23,24].

The first noteworthy finding is that beverage choices were the only significant predictor of overweight status. Children whose families reported healthier beverage consumption patterns (such as reduced intake of sugar-sweetened beverages) were considerably less likely to be overweight. This result is consistent with extensive evidence emphasizing the critical role of sugary drinks in promoting excess caloric intake, altering satiety cues, and accelerating unhealthy weight gain in childhood. Previous studies have demonstrated that reducing sweetened beverage consumption can significantly lower the risk of overweight and improve metabolic outcomes [17,25,26]. The present study reinforces this argument within the Turkish context and highlights that beverage choice may serve as an early and modifiable target for intervention, especially in school-age children.

Beyond overweight, the predictors of obesity in this study reveal a more complex behavioral pattern. Family meal frequency, family eating habits, and screen time were all significant predictors of obesity. The protective effect of frequent family meals is well-documented; shared mealtimes promote structured eating patterns, reduce impulsive snacking, and provide opportunities for parental modeling of healthy food choices [27,28,29,30,31]. Our findings support previous evidence showing that children who regularly eat meals with their families are less likely to develop obesity and more likely to adhere to healthier dietary practices.

Similarly, healthier family eating habits—reflecting balanced meals, reduced energy-dense foods, and consistent food routines—were inversely associated with obesity. This aligns with the FNPA framework and a substantial body of literature demonstrating that the home food environment profoundly influences children’s weight trajectories [9,31,32,33]. Family-level nutritional modeling, consistent meal structures, and availability of healthy options have long been considered key determinants of weight regulation.

Screen time emerged as another important predictor of obesity, supporting global research linking prolonged screen exposure to sedentary behavior, mindless eating, disrupted sleep, and increased snacking [1,34,35]. The association between screen time and obesity observed in this study underscores the multidimensional influence of digital media on children’s health, particularly in contemporary lifestyles where screen exposure begins at increasingly younger ages. Importantly, the relationship was specific to obesity rather than overweight, suggesting that screen time may play a stronger role in more advanced weight gain trajectories.

In contrast to screen time, other FNPA subdomains—including food choices, restriction/reward practices, healthy environment, family physical activity, child activity, and sleep routines—were not significantly associated with weight outcomes in this sample. This lack of association should not be interpreted as evidence that these domains are unimportant. Rather, it likely reflects methodological and contextual factors inherent to the study design. First, FNPA subdomains were assessed using parent-reported measures, which may be subject to recall bias and social desirability effects, potentially attenuating observed associations. Second, the cross-sectional nature of the study limits the ability to capture cumulative or long-term influences of behaviors such as physical activity and sleep routines, which may exert their effects over extended developmental periods rather than at a single time point. Third, the heterogeneity of family behaviors within a mall-based community sample may have further reduced the sensitivity to detect domain-specific effects. Consistent with previous research, physical activity and sleep-related behaviors may vary substantially across developmental stages and contexts, and their relationship with weight status may be indirect or mediated by other lifestyle factors [14,36,37].

Overall, the findings contribute to the pediatric obesity literature by confirming that family behaviors—particularly beverage intake patterns, shared mealtimes, structured eating routines, and screen exposure—continue to be among the most influential determinants of childhood overweight and obesity in Türkiye. These results also validate the utility of the FNPA framework in capturing modifiable home-based risk factors [10,19,38].

From a pediatric nursing perspective, the findings highlight several actionable intervention targets. Nurses working in school health, community health, and pediatric outpatient settings can play a central role in educating families about healthy beverage choices, supporting regular family meals, counseling on balanced eating habits, and guiding parents on appropriate screen-time limits. Because these behaviors are modifiable and embedded in everyday life, they represent high-impact points for preventive intervention.

Finally, this study adds to the existing literature by providing community-based data from Türkiye and emphasizes the relevance of culturally tailored, family-centered strategies in addressing childhood obesity. As a cross-sectional study, the findings are intended to inform patterns and associations rather than establish causality. Although data collection was conducted by pediatric nurses, the FNPA is a standardized, parent-reported instrument, and the findings are therefore not specific to a single professional group but may inform broader child health and obesity prevention practices.

### Strengths and Limitations

This study has several strengths. First, it included a community-based urban sample of children aged 6–17 years, which was sufficient to support the planned statistical analyses. Second, the use of the FNPA, a validated instrument capturing multiple family-level determinants of nutrition and physical activity, enabled a multidimensional assessment of factors influencing children’s weight status. Third, objectively measured anthropometric data enhanced the accuracy of overweight and obesity classification. In addition, the socio-demographic characteristics of participating families—such as parental education, employment status, and household income—were broadly comparable to those reported for urban families in Türkiye, supporting the contextual relevance of the findings for similar settings.

Despite these strengths, several limitations should be acknowledged. All FNPA and physical activity data were based on parent self-report, which is susceptible to recall and social desirability bias. The use of convenience sampling in a shopping mall may have introduced selection bias, as families visiting such settings may differ from the general population in terms of socioeconomic background, lifestyle patterns, and health behaviors. The cross-sectional design precludes causal inference, and subgroup analyses with reduced sample sizes should therefore be interpreted cautiously. Although the study was conducted in an urban setting, the findings may not fully represent children and families living in rural or semi-rural areas. Finally, the time of day at which anthropometric measurements were taken was not systematically recorded; however, any resulting within-day variation in body weight is unlikely to have meaningfully affected BMI-for-age percentile classification at the population level.

From a public health policy perspective, our finding that healthier beverage choices were associated with lower overweight risk supports the relevance of population-level strategies to reduce sugar-sweetened beverage consumption [39,40]. Fiscal measures targeting sugar-sweetened beverages have been adopted in multiple jurisdictions, including the United Kingdom (Soft Drinks Industry Levy), Mexico, South Africa (Health Promotion Levy), selected U.S. cities (e.g., Philadelphia, Seattle, and Boulder), and Middle Eastern countries such as Saudi Arabia, the United Arab Emirates, and Bahrain [41,42,43,44]. In Türkiye, certain non-alcoholic beverages are subject to excise taxation; however, unlike sugar-content–tiered models such as the UK SDIL, these taxes are not primarily structured as a sugar-density-based public health levy [45]. While our cross-sectional design does not evaluate policy effects, these findings align with the rationale of such interventions and may inform broader obesity prevention strategies.

## 5. Conclusions

This study demonstrates that specific family-level nutrition and physical activity practices—particularly beverage choices, family meal patterns, eating habits, and screen time—play an important role in determining overweight and obesity status among children in Türkiye. The findings highlight three priority behavioral targets for prevention in clinical and community pediatric nursing practice: beverage choices, family meal patterns, and screen exposure. While healthier beverage choices protected against overweight, more structured family eating routines and reduced screen exposure were associated with a lower likelihood of obesity. These findings underscore the critical role of the home environment in shaping children’s health behaviors and highlight the value of multidimensional, family-centered prevention strategies. Future longitudinal and intervention studies are needed to explore causal pathways and evaluate whether modifying FNPA-related behaviors can effectively reduce childhood obesity rates.

## Data Availability

Data are available on reasonable request from the corresponding author. The data are not publicly available due to ethical restrictions and the inclusion of sensitive data from children.
